# Querying quantitative logic models (Q2LM) to study intracellular signaling networks and cell-cytokine interactions

**DOI:** 10.1002/biot.201100222

**Published:** 2011-11-29

**Authors:** Melody K Morris, Zachary Shriver, Ram Sasisekharan, Douglas A Lauffenburger

**Affiliations:** 1 Massachusetts Institute of Technology, Department of Biological EngineeringMA, USA; 2 Cell Decision Process Center, Massachusetts Institute of TechnologyMA, USA; 3 Harvard-MIT Division of Health Sciences and Technology, Massachusetts Institute of TechnologyMA, USA

**Keywords:** Constrained fuzzy logic model, G-CSF, Multi-scale model, Signaling network

## Abstract

Mathematical models have substantially improved our ability to predict the response of a complex biological system to perturbation, but their use is typically limited by difficulties in specifying model topology and parameter values. Additionally, incorporating entities across different biological scales ranging from molecular to organismal in the same model is not trivial. Here, we present a framework called “querying quantitative logic models” (Q2LM) for building and asking questions of constrained fuzzy logic (cFL) models. cFL is a recently developed modeling formalism that uses logic gates to describe influences among entities, with transfer functions to describe quantitative dependencies. Q2LM does not rely on dedicated data to train the parameters of the transfer functions, and it permits straight-forward incorporation of entities at multiple biological scales. The Q2LM framework can be employed to ask questions such as: Which therapeutic perturbations accomplish a designated goal, and under what environmental conditions will these perturbations be effective? We demonstrate the utility of this framework for generating testable hypotheses in two examples: (i) a intracellular signaling network model; and (ii) a model for pharmacokinetics and pharmacodynamics of cell-cytokine interactions; in the latter, we validate hypotheses concerning molecular design of granulocyte colony stimulating factor.

## 1 Introduction

Based on current understanding of a biological system, bioengineers predict how the system will respond to designed perturbations. One important manifestation of this process is predicting whether exposing a patient to a drug with a pre-defined target will result in a favorable clinical outcome. This approach works well when few relevant components of the system are considered. However, it is more difficult to propagate possible effects through a complex system using intuition alone, which hinders the capability for reliable prediction.

To aid intuition, a broad spectrum of mathematical and computational models have been developed [1, 2]. For example, “theory-driven” differential equations (DEs) based on physico-chemical mechanisms have been used to model and make predictions in biological systems ranging from virus population dynamics in a host organism [3] to receptor trafficking through cellular compartments [4] to enzymatic phosphorylation cascades [5]; at the other end of the spectrum, “data-driven” algebraic and statistical algorithms have been used to understand the integrated influence of multiple signaling pathways on cell phenotypic outcomes [6, 7]. While these approaches have proven useful in biological and pharmaceutical contexts, their ability to make reliable predictions depends heavily on a large amount of appropriate experimental data for determining relationships, topologies, and parameter values. This critical dependence creates a high barrier-to-entry for using mathematical models to guide scientific decisions on a day-to-day basis. Furthermore, using these methods to describe relationships between different biological scales, such as the exchange of a molecule from tissues to individual cells and subsequent molecular interactions within the cell, is a significant challenge and an active area of research [8–10].

Logic-based models are an attractive alternative because they are readily derivable from either a theory-driven or data-driven foundation [11] and have been successfully used to predict the response of a biological system to perturbation (e.g., [12, 13]). In discrete (e.g., Boolean) logic models, all species are found categorically in one of a few levels of activity. However, this description is often too simple to adequately describe biological systems, and feedback in these models can result in oscillations which convolute interpretation of their results. Recently, some have proposed transforming discrete logic models into either ordinary or piecewise linear differential equations [14–16]. While some software tools for building and simulating models of these types exist (reviewed in [11]), changes to parameters of such models affect the differential equations governing each species, and it is not immediately evident how such changes affect the quantitative relationships among the species in the system. Moreover, use of these tools to determine the effect of perturbations to species or parameters requires familiarity with the particular software and is not straightforward.

To alleviate these difficulties, we present a new analysis framework for asking questions of logic-based models, which we term “querying quantitative logic models” (Q2LM). We use the constrained fuzzy logic (cFL) formalism recently developed for training a logic model to data [17], but here demonstrate the ability to make predictions with models based solely on prior knowledge of the biological system. Additionally, we introduce a simulation procedure that is able to solve for the steady state of a system even when feedback results in oscillatory behavior. This logic formalism allows species in a biological system to be modeled with a continuous range between zero and one using mathematical functions that directly relate input and output species (transfer functions). Importantly, the Q2LM approach facilitates querying these models for efficient prediction of the behavior of biological systems in response to perturbation. Q2LM is a MATLAB toolbox freely available at http://sites.google.com/site/saezrodriguez/software.

Because we use a simple logic-based framework, Q2LM is flexible enough to concomitantly incorporate multiple scales of biology-from molecular species to whole organisms. We illustrate the use of Q2LM to build and query a logic model with a simple example intracellular signaling model. Subsequently, we investigate a logic model of multiscale pharmacokinetics and pharmacodynamics (PK/PD) of granulocyte colony stimulating factor (G-CSF) with the objective of predicting the molecular-level alterations that would best stimulate maturation of precursor neutrophils.

## 2 Methods

### 2.1 What is a constrained fuzzy logic model?

**Figure 1:**
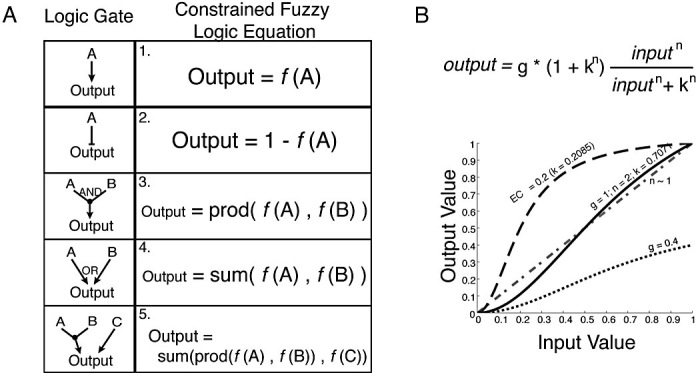
Constrained fuzzy logic. (**A**) Constrained fuzzy logic describes interactions between biological species with logic gates. The logic gates are evaluated based on the output of the transfer function (*f*) that quantitatively relates the input and output species. In this example, AND gates are evaluated with the PRODUCT operator and OR gates are evaluated with the SUM operator. Evaluation of the AND and OR gates with the MIN and MAX operators, respectively, is also supported by Q2LM. Note that the SUM operator is not identical to arithmetic sum, but rather, the logical sum of two possible values is equal to the first plus the second minus the product of the two (i.e., *V*_1_ + *V*_2_ – *V*_1_*V*_2_, where *V*_1_ is the value of one possible output and *V*_2_ is the value of the other). (**B**) The quantitative relationship between any two species is specified with a transfer function. In this paper, we use a normalized Hill function multiplied by a gain as the transfer function, although other functional forms can easily be imagined.

In a cFL model, the relationship between species is described by logic gates with transfer functions, from “upstream” parent node(s) to a “downstream” child node. In the simplest logic gate, one input parent species activates an output species, designated by an arrow between the two (Fig. 1A). In cFL, this activating relationship is represented with a transfer function, which is simply a mathematical function used to evaluate the value of the output species given the value of the input species (Fig. 1B).

In the current implementation, each transfer function is a normalized Hill function with a gain, where the gain, *g*, is a constant between zero and one, *n* is the Hill coefficient, and *k* is the parameter that determines the EC_50_ of the function. If the input species inhibits the output species (a NOT gate in traditional logic modeling, Fig. 1A), the transfer function is subtracted from one, effectively inverting it. We have found this transfer function form to be useful because it is simple yet flexible enough to accommodate a variety of biologically relevant functional relationships including linear, sigmoidal, and digital. Furthermore, each parameter of the transfer function determines a specific aspect of the function shape: *g* determines the maximum value of the output species given maximal input species value; *k* determines the EC_50_ (value of input species necessary for the output to reach activation at half of its maximum), and *n* determines whether the shape is linear or sigmoidal. Thus, changing any of these parameters changes the transfer function shape in a predictable manner (Fig. 1B).

Transfer functions are specified for every relationship between species and provide the basis for all quantitative relationships between species in a cFL model. If an output species has more than one input species, multiple transfer functions are evaluated for each input–output relationship, resulting in multiple possible values for the output species. The final value for the output species is then determined based on these possible values as well as the logic of the interactions. For example, if an output species has two inputs species, both could be necessary to affect the output species (an AND gate) or they could affect the output species independently of one another (an OR gate). If both AND and OR gates are used to relate inputs species to an output species, AND gates are evaluated before the OR gates (i.e., the sum-of-products formalism, Fig. 1A).

### 2.2 Building a cFL model

To build a logic-based model, one must first identify the species in the biological system of interest to be included in the model. These species might be intra- or extra-cellular molecules, specific cell types, or the “state” of a molecule or cell; thus, within the model a single entity can be represented by several species (e.g., ligand-bound and unbound cell receptors; differentiated or undifferentiated hematopoietic cells), where the name of the species is used to distinguish among various states of a single entity. Assigning specific names to species of any type of entity enables logic models to concomitantly incorporate processes at multiple biological scales.

The next step for building a logic model is to specify the interactions between species both in terms of the species that interact as well as whether the interaction is activating or inhibitory. Knowledge of these interactions can come from a variety of sources. An expert may have accumulated enough knowledge to build such a model using intuition alone. Additionally, a wealth of databases exists that contain such interactions [18]. It is important to document sources used during the model building process so that, if discrepancies arise between the model simulations and what is known about the system, the knowledge basis of the model can easily be revisited.

The most challenging aspect of building a logic model is specifying AND or OR logic gates for species with more than one input parent species. In previous work we used the *CellNOpt* software to train logic gates to dedicated experimental data [17, 19]. Here, we rely on prior knowledge to determine the logic of the relationships. An AND gate should be used if the input species “work together” to affect the output. Alternatively, one can identify an AND gate by asking “Should the output be affected with only that input, or are other species necessary?” If other species are necessary, an AND gate should be used. Otherwise, it is an OR gate. For example, a molecular binding event is represented with an AND gate because both binding partners are necessary to form the bound species.

The final step is to write the model in a form readable by the software. For Q2LM, this involves making a spreadsheet that specifies the interactions and parameters of the transfer functions used to evaluate the effect.

### 2.3 Simulating a cFL model

Q2LM simulates a cFL model with synchronous updating. The initial values of all non-stimuli species are designated as Not-a-Number (NaN) and ignored until their values have been specified by an upstream interacting species. At each simulation step, species' values are calculated based on the values of their input species at the previous step. Species that have been designated as “stimuli” are maintained at the stimulated value or, if its input species specify it to be a larger value during simulation, it is assigned the maximum of the stimulated and calculated values. The value of an inhibited species is multiplied by the percent inhibition at each simulation step. The simulation terminates when either the values of all species stabilize (the so-called “logic steady-state”). If any species value does not stabilize due to oscillations, the simulation will terminate after a pre-defined maximum number of steps has been reached. The value of the oscillating species can be set as a NaN, the average of several simulation steps, or calculated by solving the system of equations specifying the network.

### 2.4 Querying a cFL model

Q2LM poses the following questions: (i) “What perturbations to species in the system result in a desired outcome?” and (ii) “In what environmental conditions are these perturbations effective?” To answer these questions, one must provide environmental conditions (the “environment”), the perturbations (“experiments”) and the desired outcome (the “criteria”). Environmental conditions are considered invariant while experimental perturbations are varied and their effects within each environmental condition evaluated. Perturbation effects are then compared to the criteria to reveal if the perturbation “met” the criteria. Strictly speaking, only an environment is required to simulate the model while experiments and criteria are used to address a specific query.

### 2.5 In vivo validation of model prediction

Mice were treated with 150 mg/kg 5-fluorouracil (5FU) for 24 h prior to treatment with either wildtype or mutant colony stimulating factor for 9 days. Control mice were either treated with vehicle PBS or 150 mg/kg 5FU alone. Five mice were treated for each condition. Animals were sacrificed and blood collected by cardiac puncture. After hemolysing red blood cells using a lysis solution, white blood cells were concentrated and cell count performed with a Coulter counter. Experiments using animals we performed under the permission of MIT Committee on Animal Care protocol #0904-063-07.

## 3 Results

### 3.1 Logic-based model of an intracellular signaling network

We first exemplify the use of Q2LM with a highly simplified network that models potential crosstalk between tumor necrosis factor α (TNF-α) and transforming growth factor α (TGF-α)-induced signaling pathways. We previously observed that both TNF-α and TGF-α stimulation of HepG2 cells activated the c-Jun N-terminal kinase (JNK)/c-Jun pathway while only TGF-α stimulation activated the mitogen-activated protein kinas (MEK)/extracellular regulated kinase (ERK) pathway and only TNF-α stimulation activated the nuclear factor kappa B (NF-κB) pathway [17]. These pathways activate a variety of transcriptional programs; here, we focused on activator protein 1 (AP1) transcription factor activation, which involves the oligomerization of c-Jun and Fos. We postulated from literature evidence that ERK phosphorylates Fos, which facilitates its dimerization with c-Jun, thus forming AP1 heterodimers. Alternatively, c-Jun can be phosphorylated via the JNK pathway and dimerize to form AP1 homodimers [20–22]. To demonstrate Q2LM analysis, we question whether inhibiting the activation of MEK, ERK, or JNK would increase the amount of AP1 homodimers.

**Figure 2:**
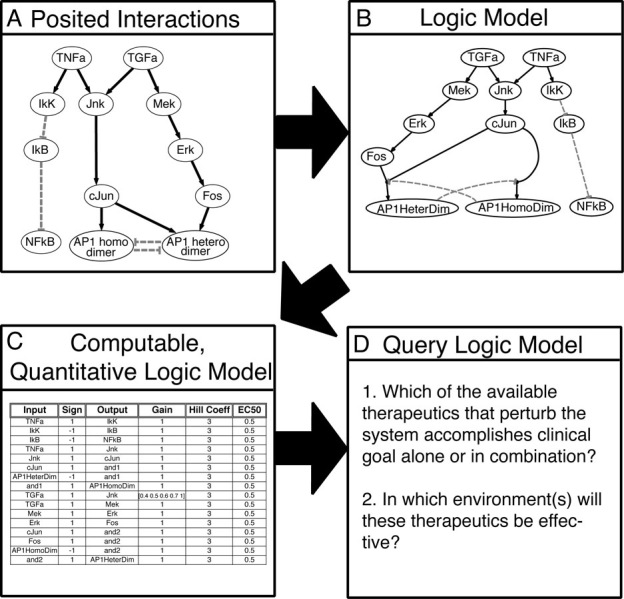
Converting posited interactions of intracellular signaling into a logic model. (**A**) The relationship between species in an intracellular signaling network is depicted graphically. Grey dashed blunted arrows indicate inhibitory interactions. (**B**) To convert the posited interactions in A into a logic model, we consider if the logic describing the relationship between input and output species should include an AND gate for species with more than one input, and find that AND gates are necessary for description of formation of AP1 homo- and heterodimers. AND gates are indicated by the input species linked to a small circle, which is further linked to the output species. (**C**) The logic model is recorded as a spreadsheet to be loaded into the Q2LM software. The first three columns specify which species interact as well as the logic of these relationships. The last three columns specify the parameters of the transfer functions of the interaction contained in that row. (**D**) Q2LM has been specifically designed to ask academically and industrially relevant questions.

From our understanding of this simple biological system, we specified the interactions between species in the network (Fig. 2A). In most cases, increasing the value of the input species increased the value, or activity, of the output species. However, there were a few cases of inhibitory interactions: IκB sequesters and inhibits the activity of NF-κB, and increased activity of IκB kinase (IκK) decreases the ability of IκB to sequester NF-κB. For this example, we also assumed that there was limited c-Jun available in the system which resulted in stoichiometry-driven inhibitory relationships between AP1 hetero- and homodimers because the presence of one dimer form indicated that there was less c-Jun available to form the other.

To convert these interactions into a logic model (Fig. 2B), we considered species with more than one parent input species for possible AND logic relating the species. Two parent inputs (TGF-α and TNF-α) activated JNK, but they did so independently of one another. Thus, this gate was an OR (not an AND gate). The AP1 heterodimers species also had more than one parent input species (c-Jun, Fos, and NOT AP1 homodimers). Because a heterodimer consists of both c-Jun and Fos, both were necessary to increase the amount of heterodimer, and an AND gate was used to model their logic. The presence of AP1 homodimer limits the amount of AP1 heterodimer, but only when c-Jun and Fos are present to make a heterodimer. Thus, it was also a parent input for the AND gate.

Finally, we wrote our logic model in a spreadsheet compatible with Q2LM (Fig. 2C). For most model parameters, we were uncertain of their values, so reasonable defaults were chosen. However, from our initial dataset, we knew that TGF-α did not activate the JNK pathway as strongly as TNF-α in HepG2 cells [17], but since we were not certain of the relative activating potentials we made several models, each with a different gain parameter for this interaction. This was indicated in the spreadsheet by including an array of gain parameters in the corresponding entry (Fig. 2C). Additionally, we added normally distributed noise to each parameter when the model was loaded to simulate biological noise.

**Figure 3:**
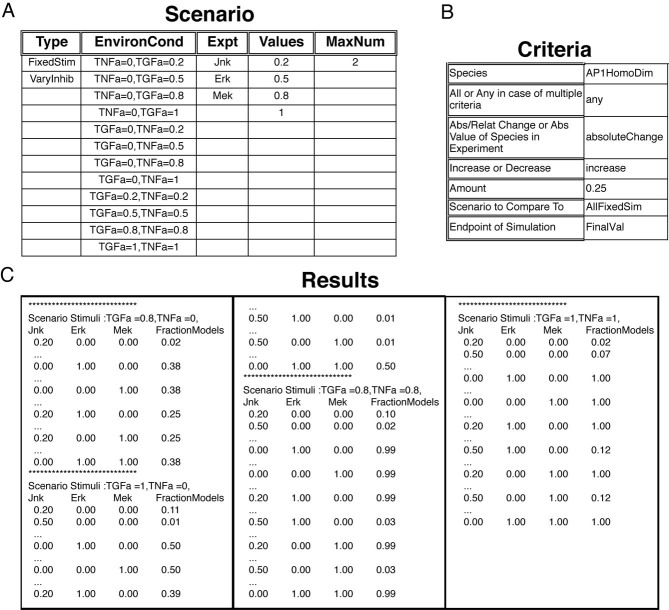
Q2LM files for examining intracellular signaling logic model. (**A**) Example of a scenario file that Q2LM imports to simulate experimental perturbations in a variety of environmental conditions. A detailed description of all file types is provided in the software's manual. In this case, environments with partial or full stimulation of TNF-α and TGF-α alone or in combination will be simulated with inhibition of the “Experimental” species JNK, ERK, and MEK at levels listed in the “Values” column alone or in combination, where the maximum number of species to inhibit at any one time is listed in the “MaxNum” column. (**B**) Example of a criteria file. Simulation results from environments with perturbation are compared to environments without perturbation and Q2LM calculates if the criteria have been met. In this case, the criterion is that the AP1HomoDim species increase in value by at least 0.25 with perturbation compared to without. (**C**) Example of portion of a Results file Q2LM outputs to indicate, for each environment, the values of perturbation that met the criteria in 3B and in what fraction of models they were effective. Ellipsis indicates conditions of intermediate doses that were not included. Tested environments for which no perturbation met the criteria are not listed.

We queried our intracellular signaling model to determine if inhibiting MEK, ERK, and JNK alone or in combination would increase AP1 homodimers in specific environments composed of varying levels of TNF-α and TGF-α alone or in combination (Fig. 2D). We simulated these environments with partial or complete inhibition of MEK, ERK, and JNK and then compared the resulting levels of AP1 homodimers with the levels that resulted without inhibition. This information was encoded in two input files: (i) the “Scenario” file included the environments and species to perturb with inhibition (Fig. 3A) and (ii) the “Criteria” file specified that the software should return experimental conditions that increase AP1 homodimers (Fig. 3B).

Q2LM results revealed perturbations that met our criteria of increased values of AP1 homodimers (Fig. 3C). We found that partially or completely inhibiting ERK and/or MEK increased AP1 homodimers in environments featuring high values of TGF-α stimulation, but had minimal effect in those with low values of TGF-α stimulation. Because this example served only to illustrate the use of Q2LM, a test of this hypothesis was out of the scope of this paper. However, we note that because the software asked questions of the model in a manner analogous to experimental queries, experimental tests are easy to specify. For this example, a follow-up experiment to test this hypothesis would be to stimulate cells with low and high concentrations of TGF-α in the presence or absence of ERK or MEK inhibition and to measure the resulting AP1 homodimer levels.

**Figure 4:**
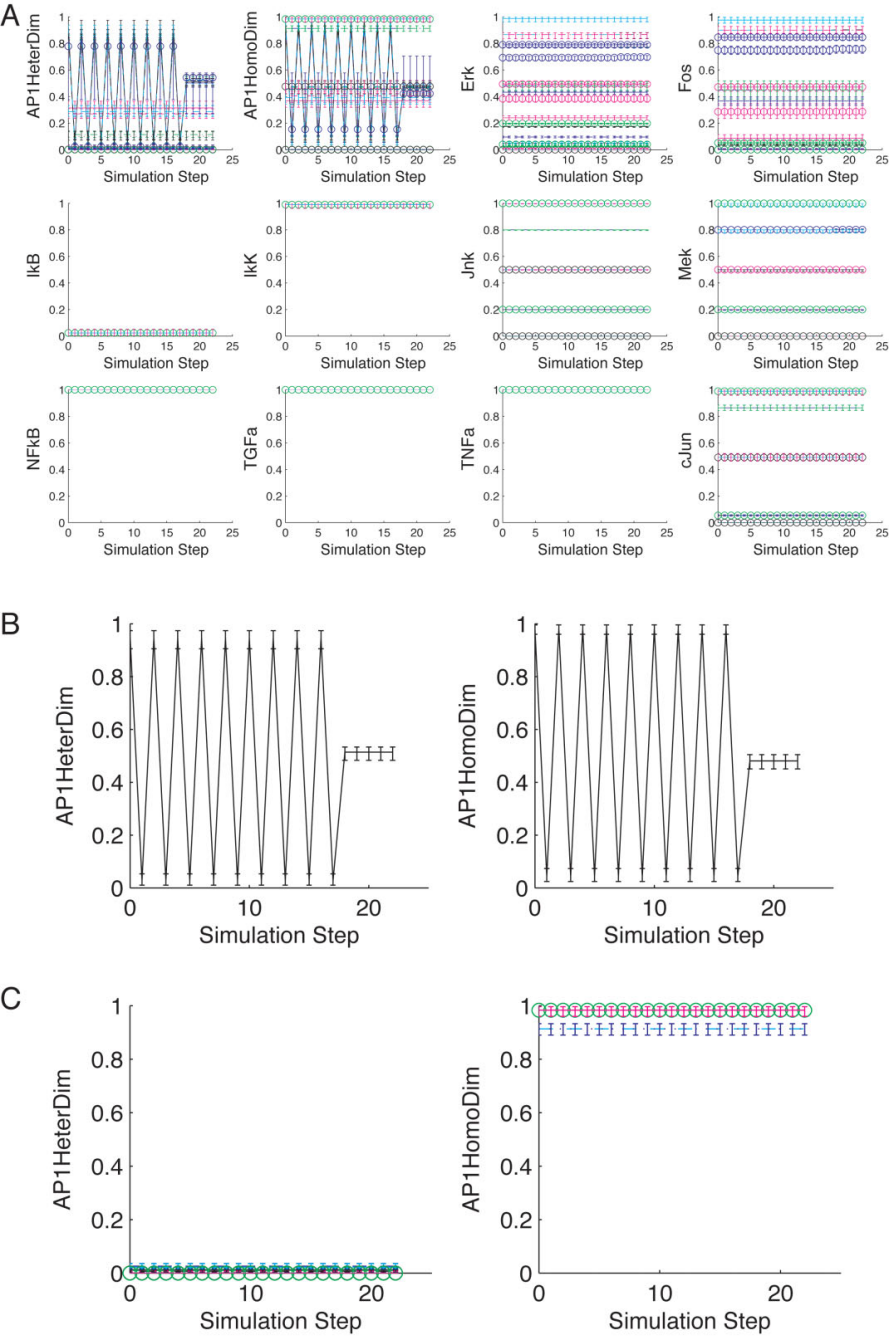
Species values as a function of simulation step for intracellular signaling model. (**A**) For each indicated species, the median value for all models at the final 19 simulation steps is shown (Q2LM does not save all simulation steps when memory is a limitation) along with the final value calculated by the solver, which has been copied several times for visualization. Upper and lower error bars indicate the third and first quartile, respectively. Simulation conditions: TGF-α = 1; TNF-α = 1; Perturbation with different combinations of JNK, MEK, and ERK inhibition is indicated by different line color. Different line styles represent different models. (**B**) Median value for AP1 homo- or heterodimers with no inhibitor perturbations. (**C**) Median value for AP1 homo- or heterodimers for inhibitor combinations that met criteria of increasing the value of AP1HomDimer by at least 0.25 in at least 25% of the models.

We next investigated how the system evolved during model simulation (Fig. 4A). It was apparent that the values of the AP1 homo- and heterodimers oscillated in several inhibition conditions. This is a common occurrence in models with feedback that have been simulated with discrete updating [14]. However, Q2LM offers a novel treatment for such cases in which the system of equations specifying the network is solved for the steady state solution of environment/perturbation combinations that exhibit oscillations (Supporting information). In this case, we observe that AP1 homo- and heterodimers oscillated due to the negative feedback between them in the absence of perturbation (Fig. 4B). However, the steady state solution of these was calculated to 0.5 because the feedback “balanced out” to an intermediate value. In inhibitor combinations that met the designated criteria, no oscillations were observed (Fig. 4C). Instead, the values of the homo- and heterodimer species approached unity and zero, respectively. Thus, these conditions increased homodimers because they were no longer limited by negative feedback from heterodimers. By examining the system evolution, we confirmed that the conditions met our criteria.

### 3.2 Logic-based modeling of pharmacokinetics of G-CSF

**Figure 5:**
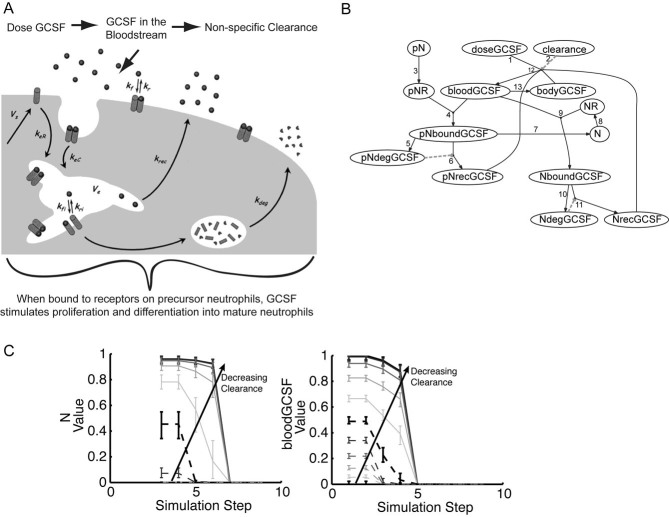
Development of logic model of G-CSF administration. (**A**) Depiction of G-CSF pharmacokinetics at the tissue, cellular, and molecular level, adapted from [23]. (**B**) Logic model based on 5A. All transfer functions have default parameters *g* = 1; *n* = 3; and EC_50_ = 0.5. Arrow labels indicate the following steps of the pharmacokinetics of the molecule: (*1*) When G-CSF is administered intravenously (*doseGCSF*), it enters the bloodstream where it is subject to (*2*) nonspecific clearance (*clearance*). (*3*) Precursor neutrophils (*pN*) possess receptors (*pNR*), which (*4*) bind G-CSF in the blood (*pNboundGCSF*). (*5*) Bound G-CSF can be degraded (*pNdegGCSF*), and (*6*) what is not degraded is recycled back into the bloodstream (*pNrecGCSF*). (*7*) Bound G-CSF also stimulates proliferation and differentiation into mature neutrophils (*N*). (*8*) Mature neutrophils possess receptors (*NR*) that can (*9*) bind G-CSF (*NboundGCSF*). Bound G-CSF is then (*10*) degraded (*NdegGCSF*) or (*11*) recycled (*NrecGCSF*). (*12*) Value of G-CSF in the blood (*bloodGCSF*) is limited by the dose, clearance, and amount recycled. (*13*) An additional species *bodyGCSF* represents the exchange of G-CSF from the blood to the body cavity and is necessary in the logic model to ensure that the *bloodGCSF* node is also limited by its own value. (**C**) The G-CSF logic model was simulated under non-limiting precursor neutrophils and dose conditions (*pN* = 1 and *doseGCSF* = 1) with multiple levels of clearance (0, 0.1, 0.2, etc.). Median value of the neutrophil and G-CSF levels in the blood nodes (*N* and *bloodGCSF*) were plotted as a function of simulation step, with error bars indicating the first and third quartile of predictions of 100 models with normally distributed noise with a standard deviation of 5% added to the transfer function parameters. As levels of clearance decreased, maximal values of *N* and *bloodGCSF* increased as well as the number of simulation steps until the species values decreased to zero. Further analysis indicated adding noise with a standard deviation of up to 25% led to identical conclusions for all results.

For our second example, we investigated whether Q2LM could be useful for multiscale models of physiological significance by using it to address the (PK/PD) of G-CSF (Fig. 5A). G-CSF is administered intravenously to stimulate the maturation of precursor neutrophils to restore neutrophil levels in situations generating neutropenia, such as chemotherapy treatment. After binding its receptor, G-CSF is internalized and either degraded in endosomes or recycled back into the bloodstream. Additionally, G-CSF is cleared from the blood through non-specific clearance mechanisms, primarily renal clearance. Sarkar et al. used a DE model of G-CSF PK/PD to predict that when non-specific mechanisms are not the dominant mechanism of clearance, decreasing the rate of endosomal degradation of G-CSF is more effective in stimulating neutrophil maturation than increasing the binding affinity of G-CSF to its receptor [23]. This insight was consistent with the effects of engineered G-CSF variants in vitro [24] but had not been verified in vivo. Here, we examined whether a simpler cFL model would allow us to reach comparable conclusions without the requirement of estimating model parameter value for a complicated mechanistic DE model.

We first converted the linguistic description of the G-CSF system above to a cFL model (Fig. 5B). Although no dedicated experimental data were used to train this model in a traditional sense, it was derived from literature knowledge describing PK/PD of G-CSF [25, 26]. Rather than using kinetic parameters to describe intracellular trafficking and non-specific clearance mechanisms, we use an AND gate to model these processes as limiting the amount of G-CSF available in the bloodstream (Supporting information). The logic description therefore allowed us to easily relate tissue level phenomena to cellular- and molecular-level phenomena.

To validate that the cFL model recapitulated known system behavior, we simulated model behavior under several conditions and plotted the species' values at each simulation step. We found that with decreasing clearance, the maximum value of both mature neutrophil (*N*) and G-CSF in the blood (*bloodGCSF*) species values increased (Fig. 5C). Although these species eventually reached a value of zero due to G-CSF degradation via receptor-mediated endocytic uptake, in some cases these decreases occurred at later simulation steps. This result agrees with how we understand the system to behave: a decrease in rate of clearance leads to an increase in total amount of G-CSF that reaches precursor neutrophils due to increased half-life, but G-CSF is nevertheless eventually cleared from the system. From this analysis, we identified two criteria to consider for assessing the impact of a perturbation on the *N* and *bloodGCSF* species: (i) maximum value attained; and (ii) the number of simulation steps during which the nodes were at a value greater than zero.

**Figure 6:**
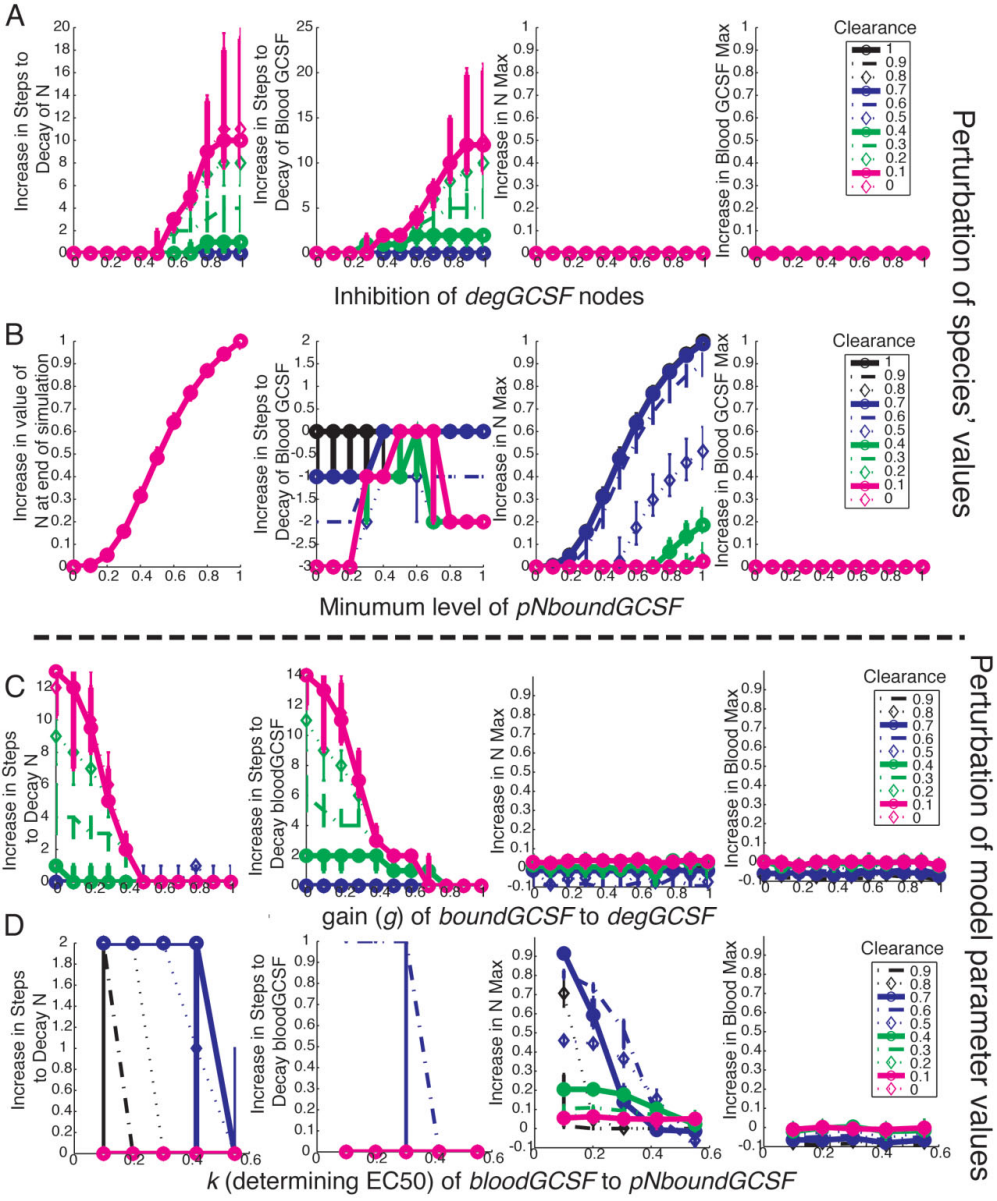
Effect of perturbations to G-CSF pharmacokinetics on criteria. In all parts, perturbations to (**A, B**) species or (**C, D**) model parameters were made when the G-CSF logic model was simulated under non-limiting precursor neutrophils and dose conditions (i.e*.*, *pN* = 1 and *doseGCSF* = 1) with multiple levels of clearance (0, 0.1, 0.2, etc.), with each color and line style corresponding to a different fixed value of the *clearance* species as shown in the legend in the rightmost panel for each part. Median effects are plotted, with error bars indicating the first and third quartile of predictions of 100 models. (**A**) The median effect of increasing inhibition of the *pNdegGCSF* and *NdegGCSF* nodes on each criteria. (**B**) The median effect of varying the minimal possible value of the *pNboundGCSF* node. Because the *N* species was not observed to decay in these simulation, the first panel is the increase in logic steady state value of *N*, instead of number of steps until decay. (**C**) The median effect of changing the gain of the transfer function relating *pNboundGCSF* to *pNdegGCSF* and *NboundGCSF* to *NdegGCSF* on each criteria. (**D**) The effect of changing the EC_50_ of the *bloodGCSF* to *pNboundGCSF* interaction.

Having established that the model was recapitulating known behavior, we explored the effects of altering G-CSF properties on physiological effectiveness, as measured by *N* and *bloodGCSF* levels. In particular, we calculated the above criteria under two conditions: (i) diminished degradation modeled by multiplying the *pNdegGCSF* and *NdegGCSF* species by a percent inhibition; or (ii) enhanced binding modeled by increasing the minimal value of the *boundGCSF* species. We then compared the values of criteria under these conditions to those from simulations with no such perturbation (Fig. 6A, B). Our results indicated that when the degradation nodes (*pNdegGCSF* and *NdegGCSF*) were inhibited by more than 50% at low values of clearance, there was a substantial increase in the number of simulation steps for the *bloodGCSF* species to reach zero. However, there was no effect on maximal value of *N* or *bloodGCSF* (Fig. 6A). On the other hand, increasing binding by setting the minimum of the *pNboundGCSF* species to a value greater than zero resulted in no decay of the *N* node (i.e., a logic steady state value greater than zero, Fig. 6B). This result was expected because the *pNboundGCSF* species directly activated the *N* species, so fixing the minimum value of one should directly affect the value of the other. This effect was also reflected in an increase in the maximum value that the *N* species attained. However, the maximal value of the *bloodGCSF* species did not increase, and in fact the number of simulation steps for the *bloodGCSF* species to reach zero decreased in many conditions (Fig. 6B). These results provided a first indication that inhibiting degradation was the better strategy for increasing numbers of mature neutrophils.

As a complementary approach, we examined the effect of varying the parameters controlling the processes of binding and degradation (Fig. 6C, D). We varied the gain parameter of the *boundGCSF*-to-*degGCSF* transfer function to represent varying the fraction of *boundGCSF* that was degraded, and found that these results recapitulated those obtained when the degradation nodes were inhibited: Steps to decay of *bloodGCSF* and *N* increased with no effect on the maximal level of these species (Fig. 6C). We also decreased the EC_50_ parameter of *bloodGCSF* to *pNboundGCSF* to represent an increase in binding affinity. By definition, decreasing the EC_50_ results in an increase in the value of *pNboundGCSF* for a given value of *bloodGCSF*. This perturbation led to a corresponding increase in maximum value of *N* while the value of *bloodGCSF* remained constant for intermediate values of clearance (Fig. 6D). At high or low values of clearance, this effect was not observed, pointing to another interesting aspect of our system: At high values of clearance, *bloodGCSF* never reached a value large enough to activate the *pNboundGCSF* and *N* nodes while at low values of clearance, the *N* species reached a large value at the default EC_50_, so only minimal effects were observed when affinity was further increased. Changing these parameter values had no substantial effects on the number of simulation steps until decay.

In summary, these results indicated that while increasing the binding affinity of G-CSF to its receptor might result in an increase in *N* for a given level of *bloodGCSF* (Fig. 6D), this effect occurred in a limited range of clearance values, and an increase in bound receptor was predicted to have the deleterious effect of decreasing the number of simulation steps required for decay of *bloodGCSF* (Fig. 6B). In contrast, decreasing the amount of degradation consistently increased the number of simulation steps required for decay of *bloodGCSF* (Figs. 6A and 6C). We therefore concluded that decreasing degradation of G-CSF is the superior strategy for stimulating neutrophil maturation.

**Figure 7:**
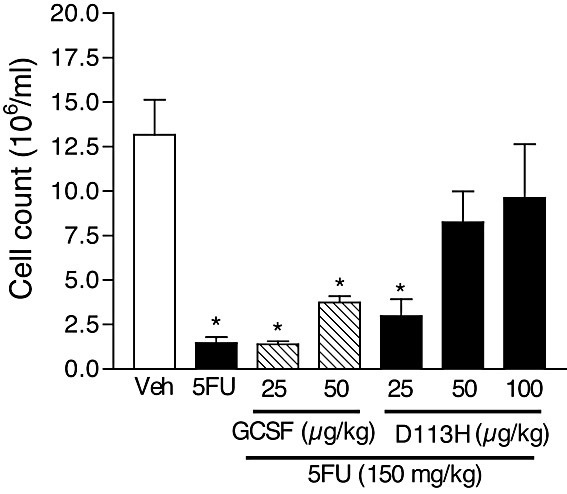
In vivoincrease in WBC associated with decreased G-CSF degradation. White blood cell count was higher in mice treated with a mutant G-CSF engineered for decreased degradation via increased dissociation in the endosomal compartment (D113H) than those treated with wildtype G-CSF. Five animals were treated with 5FU to inhibit haematopoiesis 24 h prior to treatment with the colony stimulating factor. “Veh” denotes sham treatment with PBS rather than 5FU. *p < 0.001 versus vehicle-treated controls.

Thus far, we have used in silico logic model simulations to generate hypotheses about optimization of G-CSF potency in living systems. This work suggested decreasing degradation of receptor bound G-CSF is an effective strategy for improving potency in vivo. In previous work, a mutant G-CSF with weaker receptor binding affinity at the endosomal pH exhibited decreased degradation in vitro through increased recycling of internalized receptor, resulting in increased potency of the molecule in vitro [24]. To examine whether decreased degradation had any effect in an in vivo setting, we determined white blood cell (WBC) counts in mice that were first treated with 5FU for 24 h to inhibit haematopoiesis followed by treatment with wild-type G-CSF or mutant G-CSF engineered for increased dissociation at an endosomal pH (mutant D113H), which reduces its degradation through increased recycling. In accordance with the modeling prediction, the mutant G-CSF was more effective in increasing WBC count than wild-type G-CSF (Fig. 7). This result illustrated that cFL models can faithfully represent complex multi-scale systems and that the hypotheses generated from the Q2LM analysis presented here were relevant to both in vitro and in vivo settings.

## 4 Discussion

In this work we presented Q2LM as a means for generating insights from cFL models of biological systems based on literature knowledge. We queried the models to address two questions relevant to translational research: (i) “Which therapeutic perturbation of a system will result in a pre-defined clinical goal?”; and (ii) “In which environments will these perturbations be effective?” We used this software framework to explore two biological systems of different scales. With the first, an intracellular signaling model, we illustrated use of the software to make testable hypotheses. This model exemplified several important features of Q2LM, including the ability to solve for steady state of oscillating species. In order to create an appropriately simple example, we neglected several AP1 dimer forms known to be important in physiologic response (most notably the c-Jun-ATF2 dimer [20–22]). Thus, a more complete model should be constructed to make reliable predictions regarding the affect of inhibition on AP1-mediated transcription. With the second, a multi-scale model of G-CSF administration, we generated and tested hypotheses to show that a logic model was able to recapitulate the experimentally validated results of a mechanistic ordinary differential equation without the prerequisite of estimating a multitude of kinetic parameters.

Building a logic model requires a significant amount of abstraction of the system to convert a linguistic description into logic gates. For intracellular signaling networks, this process is natural because relationships between proteins are commonly described in terms of their influence (e.g., “phosphorylation by JNK activates c-Jun” and “TGF-α stimulation activates the MEK/ERK pathway”). However, for describing interactions at the tissue, cellular, and molecular level, this process is arguably less intuitive, in part because the relationships between these types of interactions and logic gates are less obvious (e.g., it is initially unclear how “binding a receptor” and “intracellular degradation” can be described with logic gates; see Supporting information). Nevertheless, with our logic model of G-CSF we demonstrated that transforming such linguistic descriptions into a logic model can provide valuable insights into the operation of a system.

Along with abstracting the relationships between species by describing them as logic gates, the concepts of time and amount are also abstracted in a logic model. The plots presented in Figs. 4 and 5C appear similar to time courses. However, the values of species were plotted as a function of simulation step, not time. Thus, these plots allow one to directly “follow the logic” of environmental conditions and perturbations, which is not equivalent to examining the value of a species as a function of time. The exact relationship between simulation steps and time cannot be ascertained without additional information regarding the dynamic behavior of the system. Similarly, the meaning of the values of species in relation to a physical descriptor such as concentration is unclear without additional information. Nevertheless, the relative values of species in simulations of the same model carry interpretable information regarding the qualitative effect of perturbations (e.g., the value of *N* is nonzero for more simulation steps when degradation is inhibited than when it is not) that suggest a testable hypotheses (e.g., inhibiting degradation will lead to greater neutrophil maturation).

One of the main results of this work is a “seamless” approach to multi-scale modeling, exemplified by our logic model of G-CSF administration that integrates ligand/receptor binding and endocytic trafficking at the molecular level, the transition between differentiation states at the cellular level, and systemic pharmacokinetics at the tissue level. The insights from this model were validated both in vitro and in vivo. Thus, the relevance of this model to the therapeutic administration of other receptor agonists should be considered. Because intracellular trafficking is important for cellular responses to other stimulatory ligands such as epidermal growth factor (EGF) and interleukin 2 (IL-2) [27], it is likely that the insights from this model will be applicable to the administration of these molecules. More broadly, these results may be applicable to therapeutics for which endosomal degradation is an important mechanism for clearance, underscoring the importance of understanding intracellular trafficking when administering receptor agonists as therapeutics [28–30].

From this work, we submit that our Q2LM framework holds promise for effective use toward generating testable hypotheses of interest in academic and industrial settings. Additionally, the further development of cFL will enable the prediction of perturbation effects on a complex system without requiring a large amount of experimental data, thereby facilitating the use of mathematical models for guiding scientific decisions.

## Abbreviations

Q2LM: querying quantitative logic models
cFL: constrained fuzzy logic
5FU: 5-fluorouracil
G-CSF: granulocyte colony stimulating factor
WBC: white blood cell
TGF-α: transforming growth factor α
TNF-α: tumor necrosis factor α
MEK: mitogen-activated protein kinase
ERK: extracellular regulated kinase
JNK: c-Jun N-terminal kinase
AP1: activator protein 1
IκK: IκB kinase
NF-κB: nuclear factor kappa B
IL-2: interleukin 2
NaN: Not-A-Number
PK/PD: pharmacokinetics and pharmacodynamics

## Supporting Information

Detailed facts of importance to specialist readers are published as ”Supporting Information”. Such documents are peer-reviewed, but not copy-edited or typeset. They are made available as submitted by the authors.
