# Supplementary material for: Querying quantitative logic models (Q2LM) to study intracellular signaling networks and cell-cytokine interactions
Source: Biotechnol J. 2011 Nov 29;7(3):374–86. doi: 10.1002/biot.201100222 (PMC3292705; doi:10.1002/biot.201100222)
Supplement: Supplementary file 1 [file biot0007-0374-SD1.pdf]

Biotechnology
Journal

Supporting Information for DOI 10.1002/biot.201100222

Querying quantitative logic models (Q2LM) to study intracellular signaling networks and cell/cytokine interactions

Melody K. Morris, Zachary Shriver, Ram Sasisekharan and Douglas A. Lauffenburger

© 2011 Wiley-VCH Verlag GmbH & Co. KGaA, Weinheim

Supporting Text For Morris et al. Querying Quantitative Logic Models (Q2LM) to study intracellular signaling networks and cell/cytokine interactions. Biotechnology Journal

November 30, 2011

Contents

1 Comparison of cFL and other modeling formalisms **2**

2 Supplemental Experimental Methods **2**

3 Relationship between cFL and mechanistic ODEs **3**

3.1 Introduction 3

3.2 Receptor Binding 3

3.3 Receptor Degradation 6

3.4 Amount of GCSF in the blood: an example where the logic gate and mass-balance are not analogous 9

4 Simulation Procedure for Determining Steady State Value of Oscillating Species **12**

5 Supplementary References **13**

1 Comparison of cFL and other modeling formalisms

Numerous frameworks have been proposed by ourselves and others to formally train a biological network to data (e.g. artificial neural networks [1], probabilistic graphical models [2,3], and logic networks [4,5]). Our approach here is distinct from these because we base our models solely on prior knowledge using reasonable default parameters. While other frameworks could potentially be used in this manner, we argue that cFL logic models are a more attractive means for quickly and efficiently constructing a reliable model because they use logic operations that relate naturally to a linguistic description and yield interpretable results.

The observation that conclusions drawn from cFL models are abstract and species values must be considered relative to those of other species in the model points to a limitation of the technique. Thus, if the goal of a study is to predict an absolute parameter of a system (i.e. most effective Kd of a drug, recommended dose, etc.), one should use a modeling approach that is able to directly relate to physical properties (such as differential equations). However, a mechanistic differential equation model requires more precise knowledge of both the mechanisms and parameters governing system behavior. While default parameters could be assumed for DEs as we exemplified for our cFL models here, estimates for DE parameters must be at least approximately correct because different parameters regimes can yield systems with very different behavior [6]. Thus, we conclude that while cFL models are limited in that they can only make qualitative predictions, they require less precise knowledge of the system, making them an attractive alternative to mechanistic DEs.

Because the quantities resulting from cFL models are abstract, one could raise the question of whether modeling with ostensibly simpler Boolean or discrete logic would be sufficient for the analysis we present here. Indeed, cFL models use traditional AND, OR, and NOT gates to specify the topology of a network, such that tools developed for either analysis are readily interchangeable. However, the use of cFL is justified for several reasons. First, discrete models lack transfer functions such that analyses similar to that shown in main text Figures 6C and D could not easily be performed with a discrete model. Furthermore, analysis with cFL is no more difficult than one with discrete logic because of the simplicity of the cFL formalism and ease of specifying a model and its transfer functions in Q2LM. Moreover, cFL modeling allows one to explore the effects of the amount of perturbation, different implementations of perturbations, and the effect of noise in the transfer function parameters. Such explorations allow one to ascertain whether the predictions are robust to variations of the model, which if confirmed, increases confidence in their reliability.

2 Supplemental Experimental Methods

To validate the Q2LM model, we measured the ability of wild-type granulocyte colony-stimulating factor (gGCSF) or a mutant form (G43, D113H mutation) to promote hematopoiesis in 5-fluorouracil (5FU)-treated mice, similar to previously reported methods [7,8]. Briefly, B6D2F1 mice (Jackson Laboratories) were divided into seven groups of five mice each (n=35). One group of animals served as a control group and received no 5FU or colony stimulating factor. The rest of the groups were treated with 150 mg/kg 5FU for 24 hours prior to treatment with colony stimulating factor (gGCSF

or G43, injected i.p in phosphate buffered saline, supplemented with 0.1% BSA) for 9 days. Daily doses of 25 or 50 g/kg of gGCSF or 25, 50, or 100 g/kg were administered separately to a group of animals. After dosing of colony stimulating factor was completed, animals were sacrificed and blood collected by cardiac puncture. After hemolysing red blood cells using a standard lysis solution (10 mM potassium bicarbonate, 150 mM ammonium chloride, 0.1 mM EDTA, pH 8.0), white blood cells were concentrated and cell count performed with a Coulter counter (Beckman Coulter Instruments). Results are expressed in Main Text Figure 7 as an average cell count plus standard deviation.

3 Relationship between cFL and mechanistic ODEs

3.1 Introduction

While others have explored the relationship between stoichiometric maps and logic gates [9,10], these derivations point out the relationship between logic models and ordinary differential equations (ODEs) based on mass balances. A mass balance is a basic engineering concept based on the law of conservation of mass. A mass balance simply translates the statement "the rate of change of a species' mass in a defined system equals the rate of entry plus the rate of generation minus the rate of consumption and the rate of exit" into an ordinary differential equation.

The first major concept used in the derivations below is that the updating scheme in a logic model simulation is analogous to steady-state solution of an ODE. In the simulation of a logic model, each node is updated based solely on its input nodes' states at the previous time step and the concept of time is not considered. In an ODE framework, this is akin to evaluating each species as if it were at psuedo-steady state.

The figures depicting these systems (Figures 1, 3, and 6) also point out important distinctions between the interpretation of an mechanistic ODE and that of a logic model. In the graphics that motivate development of an ODE, an arrow generally indicates that the molecular species at the 'head' or 'input' of the arrow undergoes some change (i.e. is internalized, becomes bound, gets degraded, etc.). However, in a logic model, these arrows indicate only that the value of one species affects the value of another. Thus, we should understand the arrows in logic models not as indication of what happens to the input species, but rather as indications that the value of the input species (as determined by other nodes) results in some change to the output species' value.

3.2 Receptor Binding

In a mechanistic ODE modeled with mass action kinetics, a mass balance on bound receptor $[C]$ depicted in Figure 1 can be written with Equation 1:

$$\frac{d[C]}{dt} = k_f[R][GCSF] - k_r[C] - k_{endocyt}[C] \quad (1)$$

Figure 1: Binding of GCSF to its recepter. (a) graphical depiction for development of the mass balance (b) logic gate representation of this interaction

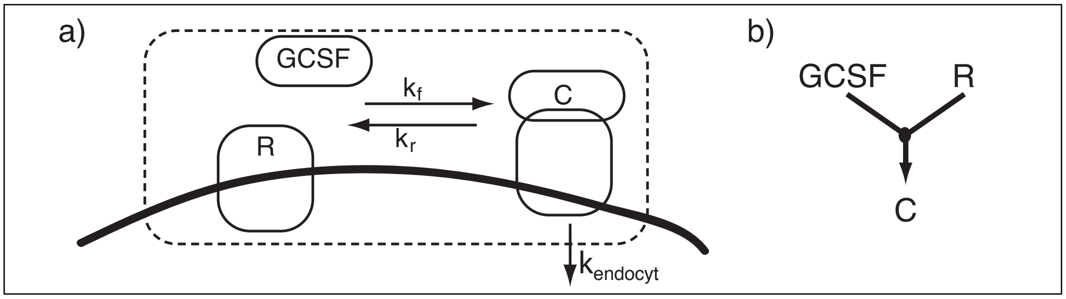

Due to the relationship between the updating scheme of a logic model and steady state described in Section 3.1, we set the derivative in Equation 1 to zero and solve for the steady state value of [C] (C_{SS}).

$$0 = k_f R_{SS} GCSF_{SS} - k_r C_{SS} - k_{endocyt} C_{SS} \tag{2}$$

$$C_{SS} = \frac{k_f}{k_r + k_{endocyt}} R_{SS} GCSF_{SS} \tag{3}$$

From Equation 3, we note that the pseudo-steady state value of [C] (C_{SS}) is a function of the product of R_{SS} and $GCSF_{SS}$. This dependence is plotted as a heat map in Figure 2a. From this plot, it is clear that this relationship between species corresponds to an AND gate in logic terms. A heat map of the Boolean logic AND gate truth table (Figure 2b) is a very abstract representation, but the cFL AND gates shown (Figure 2 c and d) demonstrate a closer relationship to the 'biochemical truth table.' Thus, we see that the AND gate relating pNR and $bloodGCSF$ to $pNboundGCSF$ is directly related to the steady-state solution of the mechanistic ODE based on mass action kinetics. Additionally, the mass-balance concept of "losing" $[C]$ due to endocytosis does not change the functional form of the relationship of its steady state value to R_{SS} and $GCSF_{SS}$.

Figure 2: Heat map of C_{SS} as a function of varying amounts of $GCSF_{SS}$ and R_{SS} make up 'Truth tables' of binding as modeled by (a) Equation 3, (b) Boolean logic AND gate, (c) cFL AND gate evaluated with the *min* operator, and (d) cFL AND gate evaluated by the *prod* operator. For evaluation of Equation 3 values of Receptor and GCSF were considered to be scaled between zero and one. For cFL evaluation, transfer functions with a gain of 1, EC_{50} of 0.5, and hill coefficient of 3 were assumed. The truth table in (a) could be directly replicated with cFL by using a linear transfer function with slope = 1 and intercept = 0.

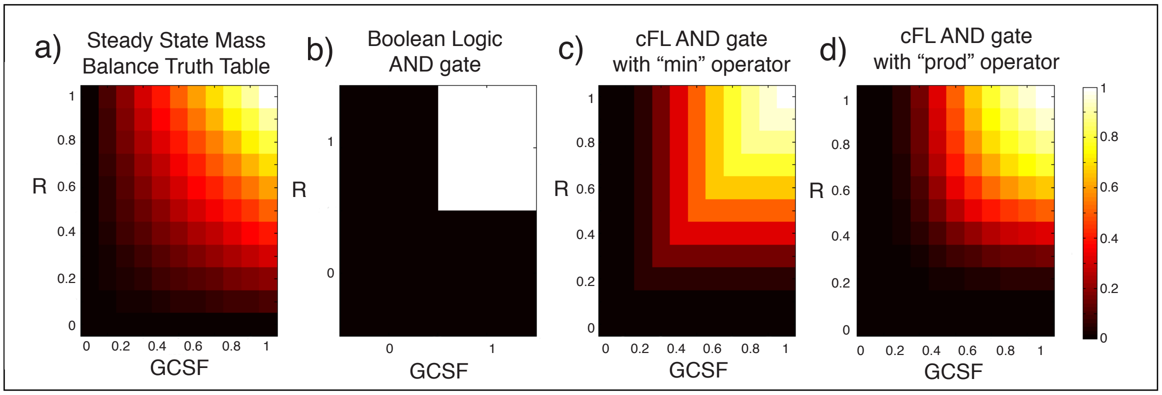

3.3 Receptor Degradation

To model endosomal degradation, we do not explicitly model all of the processes that occur mechanistically (endocytosis of both bound and unbound receptors, disassociation, etc). Rather, we use the abstract concept of "Substance" ($Subst$) shown in Figure 3 to lump all processes into one mass balance (equation 4):

$$\frac{dSubst}{dt} = Subst_{in} - Subst_{degraded} - Subst_{recycled} \tag{4}$$

Using a "fraction degraded" constant (f_{deg}) to relate $Subst_{in}$ and $Subst_{degraded}$, we obtain

$$\frac{dSubst}{dt} = Subst_{in} - f_{deg}Subst_{in} - Subst_{recycled} \tag{5}$$

Again, we use the steady state description and set the derivative in Equation 5 to zero and solve for the steady state value of $Subst_{recycled}$.

$$Subst_{recycled} = Subst_{in}(1 - f_{deg}) \tag{6}$$

From Equation 6, we again note that the steady state value of $Subst_{recycled}$ is a function of the product of $Subst_{in}$ and $(1 - f_{deg})$. In logic terms, the product again corresponds to an AND gate truth table where "1−" in $1 - f_{deg}$ indicates inhibition (Figure 4). Thus, the AND NOT gate relating $pNboundGCSF$ and $pNdegGCSF$ to $pNrecGCSF$ is related to the steady-state solution of an abstracted ODE describing the mass balance of these entities. In this case, the mass-balance concept of "losing" substance due to degradation did change the functional form of the relationship of the amount of substance recycled because this 'loss' is reflected in the logic gate by the inhibition of recycling by degradation.

Figure 3: Degradation of bound receptor. (a) graphical depiction for development of the mass balance (b) logic gate representation of this interaction

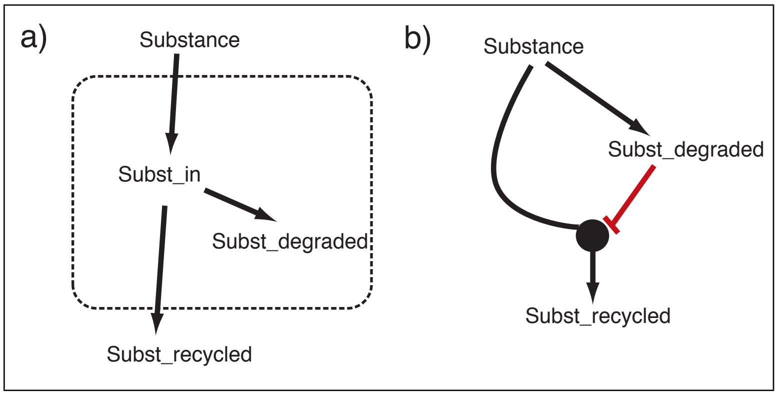

Figure 4: Heat map of $Subst_{recycled}$ as a function of varying amounts of $Subst_{in}$ and f_{deg} make up 'Truth tables' of recycling and degradation as modeled by (a) Equation 6, (b) Boolean logic AND gate, (c) cFL AND gate evaluated with the *min* operator, and (d) cFL AND gate evaluated by the *prod* operator. Equations evaluated as described in Figure 2

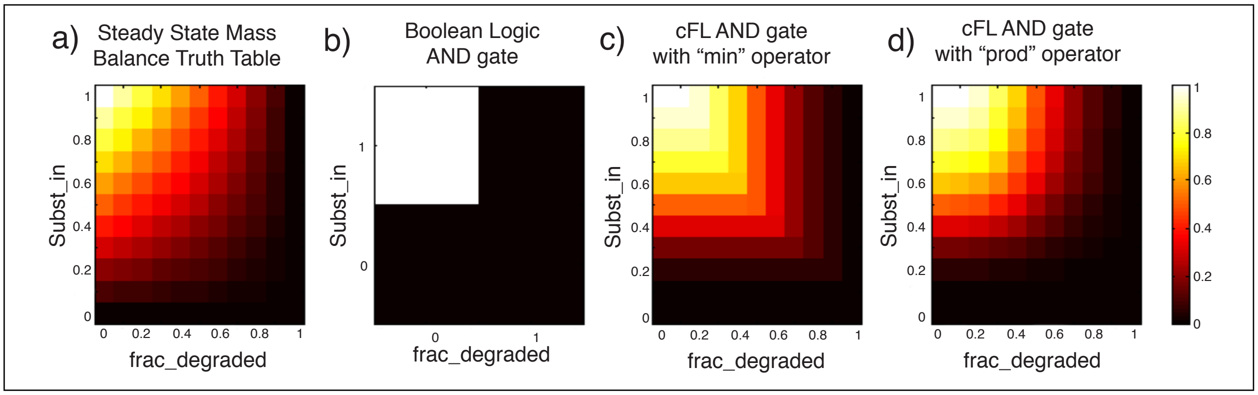

In Equation 5, we assumed that "fraction degraded" was a constant for the derivation of Equation 6. However, in Figure 3b, it is clear that the $Subst_{degraded}$ species depends on $Subst_{in}$ in the logic description. Thus, when we plot the level of $Subst_{recycled}$ as a function of only $Subst_{in}$, we notice dissimilarities in the resulting relationship (Figure 5). These dissimilarities are caused by the fact that the amount of faction of substance degraded is dependent on $Subst_{in}$ in the logic case, while it is a constant in Equation 6. It is unclear which assumption is correct in the actual biological system, and we can model the relationship specified by Equation 6 in a logic model by not having an additional 'degradation' species and instead modelling the logic as a direct interaction between $Subst_{in}$ and $Subst_{recycled}$ the 'gain' of the transfer function relating them analogous to $1 - f_{deg}$. However, this further level of abstraction hinders our ability to alter the 'degradation' species directly. Additionally, it is unclear if f_{deg} is actually independent of the amount of substance presence in the biological setting. Nonetheless, we repeated the work presented in the main text and found that the interpretation of the results is the same regardless of the logic description used for the endosomal degradation process.

Figure 5: $Subst_{recycled}$ as a function of varying amounts of $Subst_{in}$ modeled b (a) Equation 6, (b) cFL AND gate evaluated with the *min* operator, and (c) cFL AND gate evaluated by the *prod* operator. In the derivation based on mass balances (a), the variable f_{deg} is considered a constant. In those derived from the logic gate, we vary the gain of the transfer function relating $Subst_{in}$ and $Subst_{degraded}$ and consider this gain to be f_{deg}

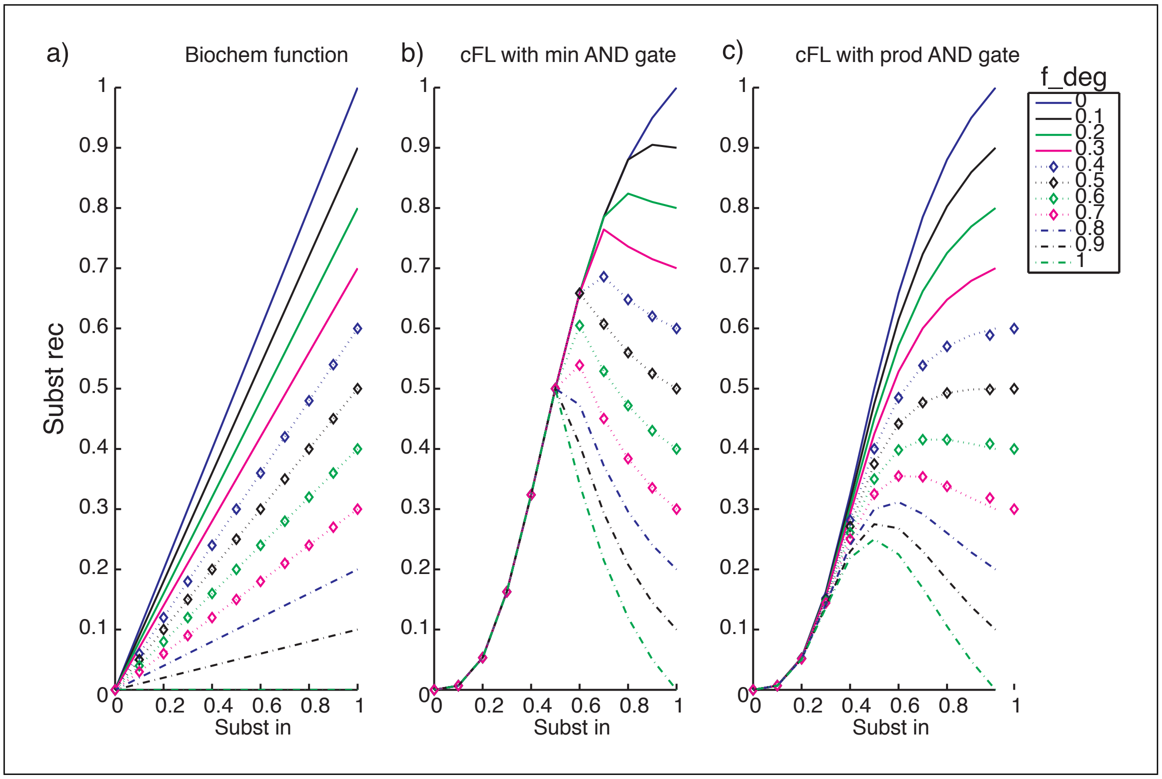

3.4 Amount of GCSF in the blood: an example where the logic gate and mass-balance are not analogous

Figure 6: Processes affecting GCSF in the blood. (a) graphical depiction for development of the mass balance (b) logic gate representation of this interaction

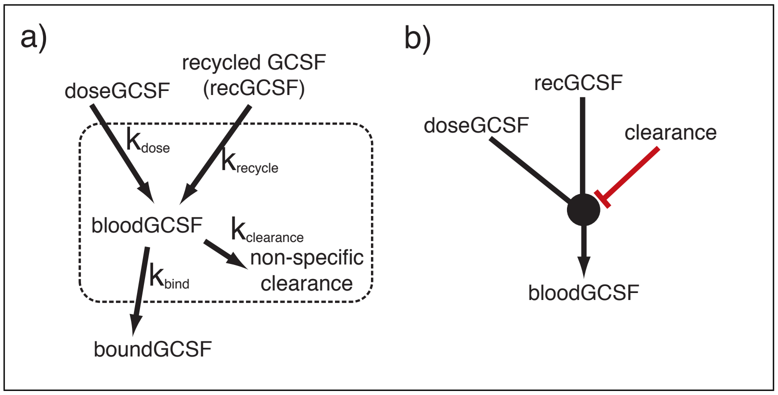

We now turn to an example where the relationship between the proper logic gate and mass-balance based ODE is not analogous. A simplified mass balance for GCSF in the blood (Figure 6) is shown in Equations 7 - 8. The 'logic' of the summation in Equation 8 would normally be an OR gate. However, in the construction of our logic model, we found that an AND gate was necessary to correctly model the logic of the bloodGCSF species because presence or absence of all of the input species to this gate can limit the value of bloodGCSF. In this case the mass balance and logic gate are not analogous. Perhaps one clue that they will not be directly related lies in Equations 7 - 8. In these equations, the two terms denoting the 'appearance' of GCSF in the blood were not dependent on a species. Rather, they were further abstracted and given as rate constants independent of other species. Additionally, the contribution of 'binding' is abstracted and modeled as simply a lumped rate rather than including the biochemical steps of association and dissociation. This abstraction at the level of rates of processes serves as an indication that this mass balance does not describe relationships between species we include in our logic model and bloodGCSF, and thus, it will not be directly relatable to our logic model.

$$\frac{dGCSF_{Blood}}{dt} = k_{dose} + k_{rec} - k_{bind}GCSF_{Blood} - k_{clearance}GCSF_{Blood} \tag{7}$$

$$GCSF_{Blood,SS} = \frac{k_{dose} + k_{rec}}{k_{clearance} + k_{bind}} \tag{8}$$

In order to correctly deduce the logic describing the bloodGCSF species, we instead turn to truth tables describing how we believe the species to relate to other species' values. We first recognize that initially, only the dose and clearance values determine the value of bloodGCSF (because at the beginning of the simulation, recycling has not yet been calculated and is thus the initial value of Not-A-Number). Thus, we will initially determine how bloodGCSF depends on the dose and

clearance species (Figure 7a) by examining the truth table for the dependence of bloodGCSF on limiting values (i.e. zero and one) of each input species (Figure 7c). We first note that dose is required for bloodGCSF to be ‘on’. Thus, we deduce that when dose is zero, bloodGCSF is also zero (Figure 7d). Next, we note that bloodGCSF is limited by clearance. Thus, we fill in the remaining two entries for the truth table (Figure 7e). This gate corresponds to an AND NOT gate (Figure 7b).

Next, we consider how bloodGCSF will depend on recycling after its value has been calculated (Figure 7f and h). The dependence on dose and clearance remains the same, so we can fill in many entries in the truth table (Figure 7i). Finally, we note that recycling is now required for bloodGCSF to remain ‘on’. Thus, we can fill in the remaining two entries of the truth table (Figure 7j) and ascertain that the recycling species should be an input to the AND gate (Figure 7g).

From this example, we see the importance of considering both the interactions between the species as well as how the species will be treated during simulation. As it is sometimes difficult to anticipate all potential factors that should be considered, we emphasize the importance of model validation at the onset of a project as well as repeatedly returning to plots of how the species’ values evolve in the course of simulation to check that no artifacts have arisen. For the GCSF example, during the course of analysis, we found that the inclusion of an additional node, ‘bodyGCSF’ (Main Text Figure 5b), was necessary in order to ensure that the model behaved properly under a few conditions where boundGCSF was fixed as a stimulation perturbation (Main Text Figure 7b). Such cases underscore the importance of model validation and highlight the benefit of being able to easily ‘follow the logic’ during model simulation to enable facile model troubleshooting.

Figure 7: Determining the logic controlling the bloodGCSF node.

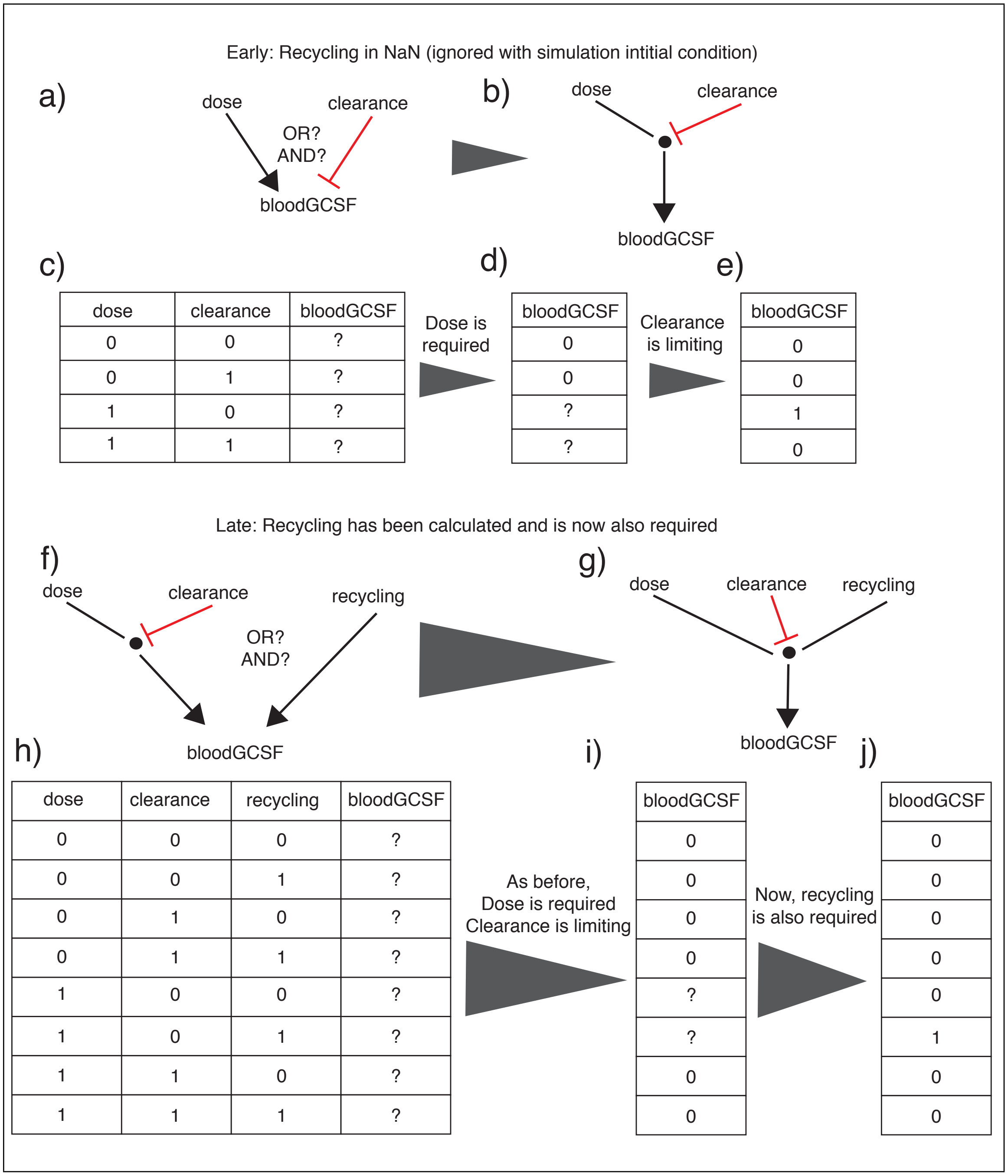

c)

dose	clearance	bloodGCSF
0	0	?
0	1	?
1	0	?
1	1	?

d)

bloodGCSF
0
0
?
?

e)

bloodGCSF
0
0
1
0

h)

dose	clearance	recycling	bloodGCSF
0	0	0	?
0	0	1	?
0	1	0	?
0	1	1	?
1	0	0	?
1	0	1	?
1	1	0	?
1	1	1	?

i)

bloodGCSF
0
0
0
0
?
?
0
0

j)

bloodGCSF
0
0
0
0
0
1
0
0

Figure 8: Simulation Procedure Pseudo-Code

```
Main Simulation:

Set initial value of species to NaN
Set stimuli values based on scenario file

While species' values haven't converged or the number of steps is less than the
maximum
    Calculate the value of all species given the value of their inputs (using transfer
        functions)
    Evaluate AND Logic
    Evaluate OR Logic
    Overwrite stimulated species' values with maximum of their simulated value or that
        given in the scenario file
    Multiply inhibited species' values by their percent inhibition
    Store species' values

Solution for oscillating species:

If a species had not converged in some condition
    For those conditions
        Set initial guess of solver to be equal to the final value for species that stabilized
            and the average of some predefined number of simulation steps for those
            that didn't stabilize
        Solve system of equations specifying network (a file with that system of
            equations is written by Q2LM for both min/max and sum/prod when the
            model is loaded)
        Store solution as final value
```

4 Simulation Procedure for Determining Steady State Value of Oscillating Species

Figure 8 describes the procedure developed to calculate the steady state of oscillating species by solving a system of equations. To solve the system of nonlinear equations for cases when species' values are observed to oscillate, we use the fsolve function in MATLAB. This function requires a default initial guess for species values. Depending on the value of the initial guess, the solver will return one of multiple possible roots. In order to return the root corresponding to the steady state of the simulation, the initial guess for each species is determined from the simulated values. Basing the initial guess on simulated species' value is key, as the solution to the equations using a default initial guess not based on simulation results can vary greatly depending on the default initial guess chosen.

5 Supplementary References

[1] Friedman, N., Inferring cellular networks using probabilistic graphical models. Science 2004, 303, 799-805.

[2] Woolf, P. J., Prudhomme, W., Daheron, L., Daley, G. Q., Lauffenburger, D. A., Bayesian analysis of signaling networks governing embryonic stem cell fate decisions. Bioinformatics 2005, 21, 741-753.

[3] Lancashire, L. J., Lemetre, C., Ball, G. R., An introduction to artificial neural networks in bioinformatics–application to complex microarray and mass spectrometry datasets in cancer studies. Brief Bioinform 2009, 10, 315-329.

[4] Morris, M. K., Saez-Rodriguez, J., Clarke, D. C., Sorger, P. K., Lauffenburger, D. A., Training signaling pathway maps to biochemical data with constrained fuzzy logic: quantitative analysis of liver cell responses to inflammatory stimuli. PLoS Comput Biol 2011, 7, e1001099.

[5] Saez-Rodriguez, J., Alexopoulos, L. G., Epperlein, J., Samaga, R., et al., Discrete logic modelling as a means to link protein signalling networks with functional analysis of mammalian signal transduction. Mol Syst Biol 2009, 5, 331.

[6] Klinke, D. J., 2nd, Signal transduction networks in cancer: quantitative parameters influence network topology. Cancer Res, 70, 1773-1782.

[7] Moore, M. A., Warren, D. J., Synergy of interleukin 1 and granulocyte colony-stimulating factor: in vivo stimulation of stem-cell recovery and hematopoietic regeneration following 5-fluorouracil treatment of mice. Proc Natl Acad Sci U S A 1987, 84, 7134-7138.

[8] Sheding, S. Media, J.E. Nakeff, A. Influence of rhG-CSF scheduling on megakaryocytopoietic recovery following 5-fluorouracil-induced hematotoxicity in splenectomized B6D2F1 mice. Stem Cells 1998, 16, 144-151.

[9] Klamt S., Saez-Rodriguez J., Lindquist J.A., Simeoni L., Gilles E. D. A methodology for the structural amd functional analysis of signaling and regulatory networks. BMC Bioinformatics 2006, 7, 56.

[10] Samaga R., Saez-Rodriguez J., Alexopoulos L. G., Sorger P. K., Klamt S. The logic of EGFR/ErbB signaling: Theoretical properties and analysis of high-throughput data. PLoS Computational Biology 2009, 5, e1000438.
